# A case with suspected ruptured mycotic coronary aneurysm presenting as purulent bloody pericarditis

**DOI:** 10.1093/ehjcr/ytag410

**Published:** 2026-06-03

**Authors:** Koji Kashiki, Kensuke Matsumoto, Akitoshi Yamada, Satoru Kawasaki, Hogara Nishisaki

**Affiliations:** Division of Cardiovascular Medicine, Department of Internal Medicine, Hyogo Prefectural Tamba Medical Centre, 2002-7, Isou, Hikami-cho, Tamba, Hyogo 669-3495, Japan; Division of Cardiovascular Medicine, Department of Internal Medicine, Hyogo Prefectural Tamba Medical Centre, 2002-7, Isou, Hikami-cho, Tamba, Hyogo 669-3495, Japan; Division of Cardiovascular Surgery, Department of Surgery, Kita-Harima Medical Centre, 926-250, Ichiba-cho, Ono, Hyogo 675-1392, Japan; Division of Cardiovascular Medicine, Department of Internal Medicine, Hyogo Prefectural Tamba Medical Centre, 2002-7, Isou, Hikami-cho, Tamba, Hyogo 669-3495, Japan; Division of Cardiovascular Medicine, Department of Internal Medicine, Hyogo Prefectural Tamba Medical Centre, 2002-7, Isou, Hikami-cho, Tamba, Hyogo 669-3495, Japan

**Keywords:** Mycotic coronary aneurysm, Purulent pericarditis, Persistent bacteraemia, Coronary artery rupture, Case report

## Abstract

**Background:**

Coronary artery aneurysms are rare, and mycotic coronary aneurysms arising secondary to bacterial infection are even rarer.

**Case summary:**

A 77-year-old woman with end-stage renal disease presented with a 1-week history of persistent fever following arteriovenous fistula creation. Transthoracic echocardiography revealed a mild pericardial effusion. On the day after admission, a blood culture yielded methicillin-sensitive *Staphylococcus aureus.* Transoesophageal echocardiography revealed a well-demarcated hypoechoic lesion encasing the right coronary artery and moderate pericardial effusion. Owing to further accumulation of pericardial effusion, urgent pericardial drainage was performed, and purulent bloody pericardial fluid was evacuated, from which *S. aureus* was isolated. Computed tomography revealed a saccular right coronary aneurysm, suggestive of a diagnosis of a ‘mycotic coronary aneurysm’ concomitant with purulent pericarditis. Despite the urgent transfer, the aneurysm ruptured before the planned surgery, and the patient subsequently died after surgical repair.

**Discussion:**

Autopsy studies have revealed that most mycotic coronary aneurysms represent ‘pseudoaneurysms’, characterized by thinning of the arterial wall with outward bulging due to disruption of the elastic tissue layers. Thus, rapid dilation of the aneurysm and eventual rupture into the pericardial cavity may be key characteristics of this pathological condition. Clinicians should maintain a high index of suspicion for mycotic coronary aneurysms in patients with persistent bacteraemia and pursue prompt multimodal imaging for early detection, particularly when the standard evaluations for infective endocarditis are unknown. Furthermore, in the presence of haemorrhagic pericardial effusion, timely surgical intervention is imperative, because it indicates aneurysmal rupture.

Learning points

- Clinicians should pursue prompt multimodal imaging for early detection, particularly when the standard evaluations for persistent bacteraemia are unknown.
- Timely surgical intervention is imperative in the presence of haemorrhagic pericardial effusion, which is an ominous indicator of aneurysmal rupture.

## Introduction

Coronary artery aneurysms are rare, with a reported prevalence of <1% in the general population.^1^ The aetiology encompasses atherosclerosis, connective tissue disorders, congenital abnormalities, sequelae of Kawasaki disease, traumatic coronary injury, and iatrogenic causes following percutaneous coronary intervention.^2^ By contrast, ‘mycotic coronary aneurysms’—those arising secondary to bacterial infection—are extremely rare.^3–9^

Herein, we present a highly unusual case of a patient with end-stage renal disease who developed *Staphylococcus aureus* bacteraemia following the creation of an arteriovenous fistula, which was subsequently complicated by findings suggestive of a mycotic coronary artery aneurysm and purulent pericarditis.

## Summary figure

**Figure:**
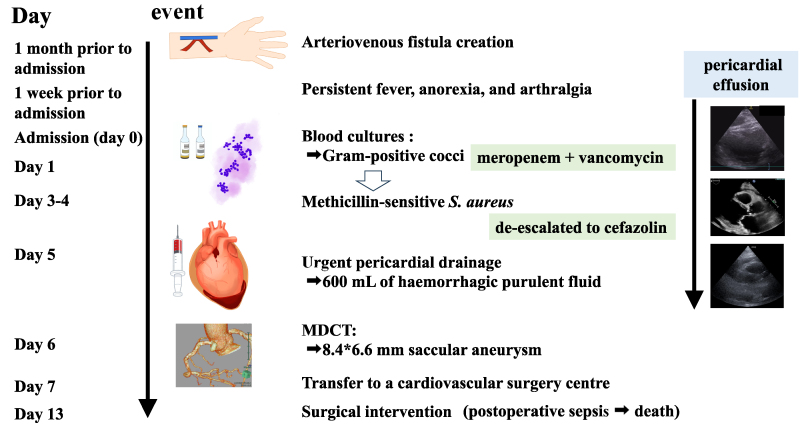


## Case presentation

A 77-year-old woman presented with a 1-week history of persistent fever, appetite loss, and arthralgia. Her medical history included an old myocardial infarction and end-stage renal disease. Approximately 1 month prior to presentation, an arteriovenous fistula was created in her right forearm in preparation for imminent haemodialysis; however, the patient had no apparent episode of bacteraemia or wound infection following the surgical procedure. At the emergency department, her vital signs were stable, with a blood pressure of 112/52 mmHg and heart rate of 68 b.p.m., although her body temperature was elevated at 38.2°C. Physical examination revealed no conjunctival petechiae, Janeway lesions, or Osler nodes in the extremities. Auscultation revealed a Levine grade II/VI systolic ejection murmur; however, no pericardial friction rub was appreciated. Inspection of the surgical site revealed no signs of infection at the arteriovenous fistula, and ultrasonographic evaluation demonstrated no evidence of infectious complications such as septic thrombophlebitis or a mycotic aneurysm. Chest radiography revealed water bottle–shaped cardiomegaly with slight bilateral pleural effusion (*Figure 1A*). Twelve-lead electrocardiography revealed a normal sinus rhythm and poor R-wave progression from leads V1 to V4; however, PR segment depressions or ST-segment elevations suggestive of pericarditis were absent (*Figure 1B*). Laboratory findings revealed elevated inflammatory markers, with a white blood cell count of 14 000/μL (normal range: 3500–9000/μL) and a C-reactive protein level (normal range: <0.3 mg/dL) of 29.8 mg/dL. However, no other laboratory abnormalities suggestive of a specific source of infection were identified. In the emergency department, transthoracic echocardiography demonstrated reduced left ventricular systolic function with an ejection fraction of 31.3%, along with wall motion abnormalities localized to the anteroseptal wall, consistent with a history of myocardial infarction (see Supplementary material online, *Movie S1*). Newly identified mild pericardial effusion was observed (1–2 mm; Supplementary material online, *Movie S2*). The patient was admitted for further evaluation of an acute febrile illness of unknown origin, and empirical antimicrobial therapy with meropenem and vancomycin was initiated. On the third day after admission, blood cultures yielded methicillin-sensitive *S. aureus* (MSSA) from all four blood culture bottles, prompting transoesophageal echocardiography on Day 4 for further evaluation. Although no infective vegetation or annular abscesses were observed, transoesophageal echocardiography revealed a well-demarcated hypoechoic lesion encasing the aneurysmal right coronary artery (coronary aneurysm, 5.5 × 4.6 mm; hypoechoic lesion, 15 × 16 mm; *Figure 2A*; Supplementary material online, *Movie S3*). Moreover, a moderate amount of pericardial effusion was observed (8 and 10 mm; *Figure 2B*; Supplementary material online, *Movie S4*), which had increased in volume compared with that at initial presentation. On Day 5 of hospitalization, further accumulation of pericardial effusion was observed (10 and 20 mm; Supplementary material online, *Movie S5*), which prompted urgent pericardial drainage. A total of 600 mL of purulent and bloody fluid was drained by pericardiocentesis. Although viral, autoimmune, tuberculous, and malignant pericarditis were considered potential causes of pericardial effusion accompanied by fever, cytological examination revealed numerous neutrophils with prominent phagocytosis of MSSA, leading to a diagnosis of bacterial pericarditis. From Day 4 onwards, antimicrobial therapy was de-escalated from empirical meropenem and vancomycin to cefazolin (2 g/day), based on antimicrobial susceptibility testing and the patient’s renal function, and was continued thereafter. Multidetector-row computed tomography (MDCT) performed on Day 6 revealed a haematoma located in the atrioventricular groove between the right atrium and right ventricle (7.2 × 6.6 mm for the coronary aneurysm and 32 × 30 mm for the haematoma; *Figure 3*; Supplementary material online, *Movie S6*) accompanied by pericardial effusion. Furthermore, the volume-rendered image clearly depicted a saccular aneurysm arising from the ostium of the right coronary artery (*Figure 4*), with accompanying findings that could not rule out contained rupture (*Figure 5*, arrowhead). Based on these comprehensive multimodal investigations, impending rupture of a coronary artery aneurysm suggestive of a mycotic aetiology, along with purulent pericarditis likely secondary to MSSA bacteraemia, was considered. On Day 7, the patient was urgently transferred to a cardiovascular surgery centre for urgent surgical intervention. Cefazolin was continued until surgical intervention.

**Figure 1:**
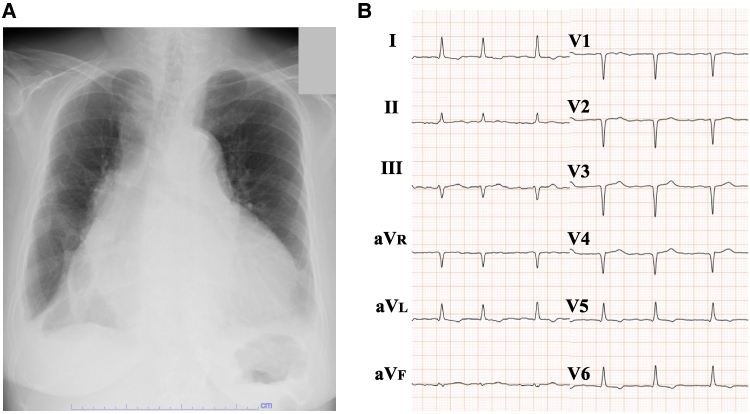
Chest radiograph and electrocardiogram of a 77-year-old woman. (*A*) Chest radiograph showing water bottle–shaped cardiomegaly with bilateral pleural effusion. (*B*) Twelve-lead electrocardiogram demonstrating normal sinus rhythm, poor R-wave progression from leads V1 to V4, and T-wave inversions in leads I, aVL, and V5–V6. However, there were no PR segment depressions nor ST segment elevations suggestive of pericarditis.

**Figure 2:**
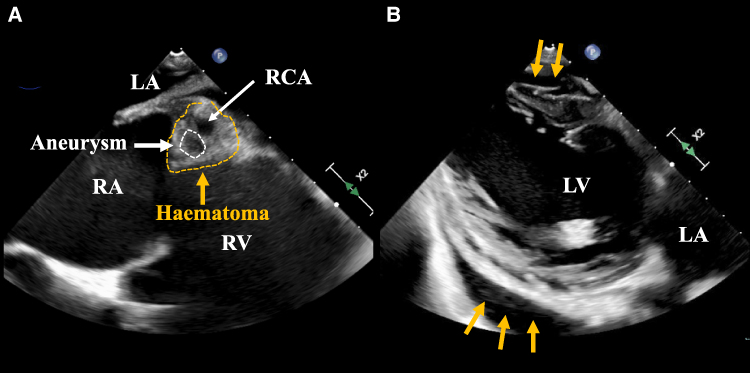
Transoesophageal echocardiography findings of a 77-year-old woman. (*A*) Modified mid-oesophageal right ventricular inflow view revealing a well-demarcated, low-echogenic lesion surrounding the right coronary artery, suggesting a mycotic aneurysm (5.5 × 4.6 mm for the coronary aneurysm and 15 × 16 for the hypoechoic lesion; the hypoechoic lesion is outlined by standard [black] dots). (*B*) Transgastric two-chamber view demonstrating moderate amount of pericardial effusion (standard arrows) surrounding the left ventricle. RA, right atrium; RV, right ventricle; LA, left atrium; LV, left ventricle; RCA, right coronary artery.

**Figure 3:**
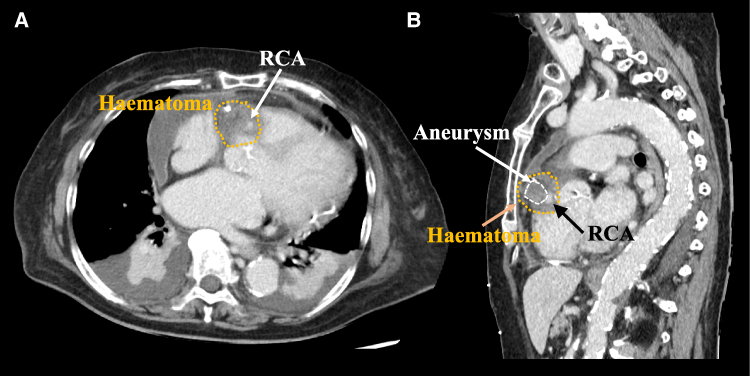
Contrast-enhanced computed tomography of a 77-year-old woman. (*A*) Axial image showing a low-attenuation area located in the atrioventricular groove between the right atrium and right ventricle (outlined by standard dots), accompanied by a pericardial effusion, suggesting a mycotic aneurysm with purulent pericarditis. The size of aneurysm was measured as 7.2 × 6.6 mm for the coronary aneurysm and 32 × 30 for the surrounding haematoma; the haematoma is outlined by standard dots. (*B*) Sagittal image further illustrating the spatial relationship between the aneurysm (the haematoma is outlined by standard dots) and adjacent cardiovascular structures, confirming its location along the course of the right coronary artery. RCA, right coronary artery.

**Figure 4:**
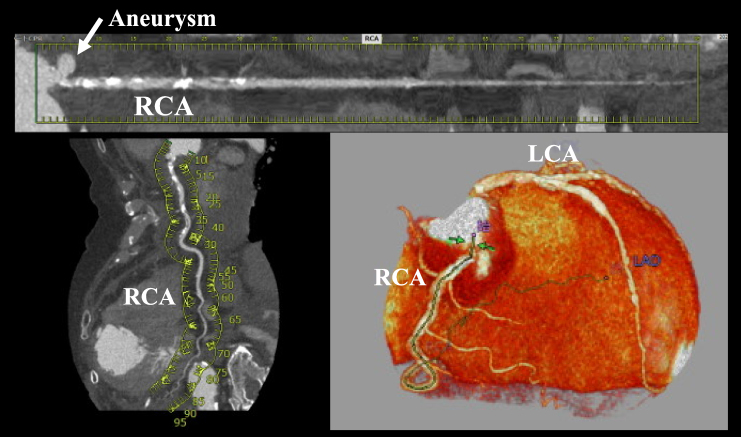
Stretched curved planar reconstruction image from multidetector-row computed tomography of the right coronary artery. A stretched curved planar reconstruction image of the right coronary artery clearly depicts the anatomical relationship between the saccular aneurysm and right coronary artery. LCA, left coronary artery; LAD, left anterior descending artery; RCA, right coronary artery.

**Figure 5:**
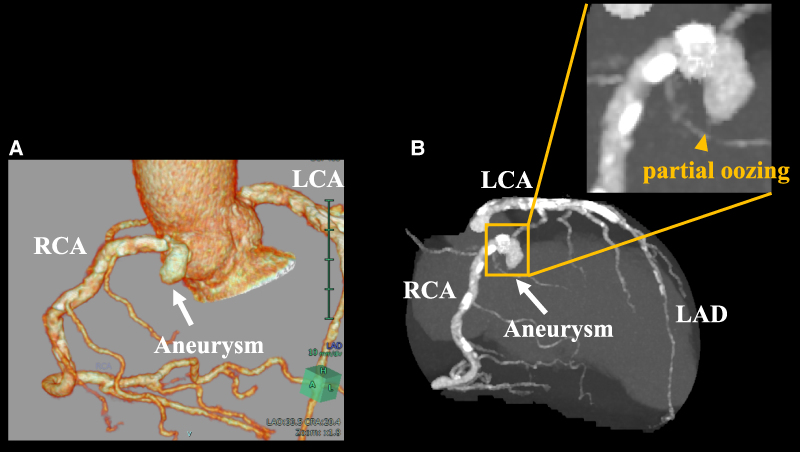
Multidetector-row computed tomography of the coronary arteries. (*A*) Volume-rendered three-dimensional image clearly depicting a saccular aneurysm (arrow) arising from the ostium of the right coronary artery. (*B*) Maximum intensity projection image of the right coronary artery clearly showing the location and size of the aneurysm. Furthermore, contrast extravasation is observed at the tip of the saccular aneurysm (arrowhead), suggesting a subsequent oozing rupture. LCA, left coronary artery; LAD, left anterior descending artery; RCA, right coronary artery.

Thirteen days after the initial admission, although open-heart surgery was initiated, the patient experienced cardiac arrest immediately after thoracotomy. Intraoperative findings revealed a large amount of purulent fluid within the pericardial cavity and dense adherence between the pericardium and myocardium. Given the urgent nature of the procedure, no pathological specimens were submitted for histological examination. However, intraoperative inspection confirmed that the ostium of the right coronary artery was covered with fragile, infected tissue accompanied by a haematoma and had been destroyed by infection, suggesting contained rupture of the right coronary artery pseudoaneurysm. Despite receiving an adequate dose of cefazolin continuously for 17 days, from antimicrobial de-escalation prior to surgery until her death, in addition to patch repair and coronary artery bypass grafting, the patient ultimately succumbed to sepsis on the seventh post-operative day.

## Discussion

Mycotic coronary artery aneurysms are exceptionally rare and are most often observed in immunocompromised individuals as a complication of infective endocarditis or bacteraemia.^3–8,10^ These aneurysms account for <3% of all coronary artery aneurysms and occur in <0.5% of patients with infective endocarditis.^3,11^ They may be complicated by bacterial pericarditis^5–7^ and, in some cases, by progressive aneurysmal dilatation or rupture, making it a potentially lethal condition.^5–7,9,10^ The underlying pathogenesis is presumed to involve bacterial seeding at sites of pre-existing atherosclerotic lesions or injured endothelium, leading to infectious aneurysm formation.^12^ *S. aureus*, as identified in the present case, is responsible for the majority of mycotic coronary artery aneurysms.^12^

Although coronary angiography,^6,7^ cardiac MDCT,^3,5,8–10^ or magnetic resonance imaging^6,12^ can provide important diagnostic information in suspected mycotic artery aneurysms, definitive diagnosis ideally requires histopathological confirmation. In the present case, although histopathological confirmation was not available because no specimen was submitted for pathological examination during the emergent surgery, multimodal imaging findings, together with MSSA bacteraemia, purulent haemorrhagic pericardial effusion, and intraoperative evidence of infected destruction of the right coronary ostium, strongly supported the presumptive diagnosis. Cardiac MDCT is particularly advantageous for delineating the coronary anatomy, including the location and size of aneurysms and coexistent stenotic lesions. Moreover, MDCT can provide extravascular information, facilitate the diagnosis of pseudoaneurysms, and characterize anatomical relationships with adjacent structures.^8–11^ It is especially useful in assessing the presence and amount of pericardial effusion, extension of infection to the adjacent structures, including the annulus and myocardium, and planning for surgical intervention.

In this case, whether a suspected mycotic aneurysm first developed and subsequently ruptured, leading to secondary purulent pericarditis, or whether bacterial pericarditis preceded the formation of the aneurysm via the contiguous spread of infection remains unclear. However, pericardial fluid obtained via pericardiocentesis was purulent and grossly haemorrhagic. Thus, the aneurysm, which formed against a background of severe atherosclerosis in an immunocompromised host, may have developed secondary to *S. aureus* bacteraemia and subsequently ruptured into the pericardial space, resulting in purulent and bloody pericarditis.

If not appropriately diagnosed and promptly treated, mycotic coronary artery aneurysms often follow an invariably fatal course, with reported mortality rates of 40%–50% due to sepsis or aneurysmal rupture.^3,13^ Autopsy studies have revealed that most mycotic coronary artery aneurysms represent ‘pseudoaneurysms’, characterized by thinned arterial wall bulging outward due to disruption of the elastic tissue layers.^14^ Thus, rapid and irreversible dilation of the aneurysm^7^ and eventual rupture into the pericardial cavity^5,10,11^ appears to be a key characteristic of this pathologic condition. Therefore, urgent surgical resection combined with coronary artery bypass grafting remains the primary treatment.^6–9,15^ This is particularly critical in cases where rupture is imminent, such as in the presence of bloody pericardial effusion, as observed in this case, or if it has already occurred. Because a post-mortem autopsy was not performed due to lack of consent from the family, the exact cause of death remains unclear in this case. However, based on the clinical context, in the setting of a substantial pericardial effusion with elevated intrapericardial pressure and uncontrolled sepsis, the vasodilatory effects of general anaesthesia may have been superimposed, leading to profound systemic hypotension, critically reduced perfusion pressure to vital organs, and ultimately a fatal outcome.

From a diagnostic perspective, the differential diagnosis of persistent bacteraemia encompasses six principal pathological entities: infective endocarditis, catheter-related bloodstream infection, thrombophlebitis, cardiac implantable electronic device infection, vascular graft infection, and mycotic aneurysms. Accordingly, each possibility must be rigorously evaluated and excluded based on the patient’s clinical background, physical examination, ultrasonographic evaluation, and radiological findings. Therefore, clinicians must maintain a high index of suspicion for mycotic coronary artery aneurysms in immunocompromised patients. In particular, when standard investigations for persistent bacteraemia yield negative results, mycotic coronary artery aneurysms should be included in the differential diagnosis.^6^ Furthermore, because mycotic coronary aneurysms are essentially pseudoaneurysms,^14^ urgent surgical intervention is mandatory, especially in cases presenting with haemorrhagic pericardial effusion indicative of imminent rupture,^10^ as exemplified by the present case.

## Conclusion

We report a rare case of a suspected mycotic coronary artery aneurysm complicated by purulent pericarditis following *S. aureus* bacteraemia in a patient with end-stage renal disease. Unfortunately, the patient succumbed to aneurysmal rupture immediately prior to surgical intervention. Prompt and accurate diagnosis using multimodal imaging, followed by timely aneurysmectomy, is essential for improving patient outcomes.

## Supplementary Material

ytag410_Supplementary_Data

## Data Availability

Data is accessible upon reasonable request; however, the authors reserve the right to individually assess and decide upon each request.

## Lead author biography

Koji Kashiki graduated from Kobe University Graduate School of Medicine, Japan, in 2017. After completing residency, he is currently a general physician with a major in family medicine at Tamba Prefectural Medical Centre.

## Notes

**Consent:** Written consent for the submission and publication of this case report, including all images and associated text, was obtained from the patient in accordance with COPE guidance.
